# Quantifying the impact of chronic conditions on a diagnosis of major depressive disorder in adults: a cohort study using linked electronic medical records

**DOI:** 10.1186/s12888-016-0821-x

**Published:** 2016-04-26

**Authors:** Euijung Ryu, Alanna M. Chamberlain, Richard S. Pendegraft, Tanya M. Petterson, William V. Bobo, Jyotishman Pathak

**Affiliations:** Department of Health Sciences Research, Mayo Clinic, Rochester, MN USA; Department of Psychiatry and Psychology, Mayo Clinic, Rochester, MN USA; Division of Health Informatics, Department of Healthcare Policy & Research, Weill Cornell Medicine, Cornell University, New York, NY USA

**Keywords:** Major depressive disorder, Depression, Multimorbidity, Comorbidity, Risk factors, Relative influence

## Abstract

**Background:**

Major depressive disorder (MDD) is often comorbid with other chronic mental and physical health conditions. Although the literature widely acknowledges the association of many chronic conditions with the risk of MDD, the relative importance of these conditions on MDD risk in the presence of other conditions is not well investigated. In this study, we aimed to quantify the relative contribution of selected chronic conditions to identify the conditions most influential to MDD risk in adults and identify differences by age.

**Methods:**

This study used electronic health record (EHR) data on patients empanelled with primary care at Mayo Clinic in June 2013. A validated EHR-based algorithm was applied to identify newly diagnosed MDD patients between 2000 and 2013. Non-MDD controls were matched 1:1 to MDD cases on birth year (±2 years), sex, and outpatient clinic visits in the same year of MDD case diagnosis. Twenty-four chronic conditions defined by Chronic Conditions Data Warehouse were ascertained in both cases and controls using diagnosis codes within 5 years of index dates (diagnosis dates for cases, and the first clinic visit dates for matched controls). For each age group (45 years or younger, between 46 and 60, and over 60 years), conditional logistic regression models were used to test the association between each condition and subsequent MDD risk, adjusting for educational attainment and obesity. The relative influence of these conditions on the risk of MDD was quantified using gradient boosting machine models.

**Results:**

A total of 11,375 incident MDD cases were identified between 2000 and 2013. Most chronic conditions (except for eye conditions) were associated with risk of MDD, with different association patterns observed depending on age. Among 24 chronic conditions, the greatest relative contribution was observed for diabetes mellitus for subjects aged ≤ 60 years and rheumatoid arthritis/osteoarthritis for those over 60 years.

**Conclusions:**

Our results suggest that specific chronic conditions such as diabetes mellitus and rheumatoid arthritis/osteoarthritis may have greater influence than others on the risk of MDD.

## Background

Major depressive disorder (MDD) is highly prevalent, both in the US and worldwide. Among US adults, the estimated 12-month and lifetime prevalence rates are 8.3 and 19.2 %, respectively, and MDD is twice as common in women as in men [1, 2]. The World Health Organization (WHO) considers MDD to be the third-highest cause of disease burden world-wide, and the highest cause of disease burden in the developed world [3]. MDD is associated with declines in overall health that are equivalent to cardiovascular disease, diabetes, and arthritis, and the presence of chronic disease with MDD contributes to greater disease burden and cost to society than that of each illness alone [4].

Comorbidity refers to the presence of at least one extra chronic disease along with a chronic disease of interest, and is known to be common at all ages, but especially among the elderly. Several studies have reported high rates of MDD co-occurrence with many chronic somatic conditions including cardiovascular diseases, cancer, and diabetes [5–8]. MDD patients with comorbid chronic somatic conditions tend to have worse depression outcomes than patients without such comorbidities, including more persistent depressive symptoms, higher relapse rates, longer time to recovery [9–11], and greater utilization of medical resources [12]. These observations may be due to negative reciprocal interactions between MDD, depressive symptoms, and chronic somatic illness. Pain and functional impairment associated with chronic conditions may increase the risk of MDD, but MDD may also exacerbate the pain and distress associated with somatic illnesses (NICE guidelines [CG91] Published date: October 2009), suggesting a bi-directional relationship between MDD and chronic somatic illness.

The majority of studies examining the relationship between MDD and chronic conditions have focused on assessing MDD prevalence for a given somatic condition, which does not permit the examination of specific chronic conditions as risk factors for MDD in the context of other co-occurring physical illnesses. Consideration of the comparative contributions of specific chronic somatic conditions to MDD risk in the context of all co-occurring conditions is important, given that patients with chronic illnesses tend to have more than one such condition, particularly if they are depressed [8].

The age of MDD onset is highly variable. Community surveys have suggested that incidence peaks occur during ones’ 20’s and 50’s [13], although incident MDD can first present at virtually any point across lifespan [14]. More recent data suggest that a substantial number of cases present very early in life, i.e., during childhood or adolescence [15]. In addition to MDD onset, age is also strongly associated with the types of commonly occurring chronic conditions [16]. On that basis, it is possible that patterns of co-occurring conditions will vary in MDD patients depending on the age of MDD onset.

In this study, we aimed to investigate the association of selected commonly-occurring chronic conditions on the risk of MDD onset depending on age, and to quantify the relative importance of each chronic condition on MDD risk in the presence of all remaining physical conditions, rather than examining each condition separately. In doing so, we utilized electronic health record (EHR) data for ascertaining MDD disease status and co-occurring chronic conditions, offering the advantage of studying a large population of over 11,000 MDD cases and an equal number of patients without MDD. We hypothesized that the somatic conditions with the greatest relative importance for MDD differ according to age.

## Methods

### Study subjects

In this study, we considered all adult (aged ≥ 18 years) patients empanelled with primary care in June, 2013 at Mayo Clinic, Rochester, MN. These patients mostly resided in Olmsted County, MN and the eight neighboring southeast MN counties. EHRs were used to construct the cohort and to ascertain the variables for this study. The EHR data comprises longitudinal records of structured data (e.g., diagnoses, procedures, and laboratory measurements) and unstructured text (e.g., clinical notes, and pathology reports).

### Subjects with newly diagnosed MDD

Cases of MDD that were newly diagnosed between 2000 and 2013 were identified using a validated EHR-based algorithm [17]. Validation of this algorithm was conducted prior to the current study. Briefly, the EHR-based algorithm used to identify newly diagnosed MDD cases was validated using data from 6923 subjects from the Mayo Genomic Consortium, a cohort of Mayo Clinic patients with EHR-linked data [18]. The algorithm identified 836 patients (12 %) with MDD. Of these, 82.3 % were confirmed as being true cases of newly diagnosed MDD using manual chart review as a gold standard. All patients that did not meet the electronic algorithm definition of newly diagnosed MDD were confirmed by record review as being non-cases.

For the current study, MDD cases were identified on the basis of having met at least two of the following criteria: ≥ 2 MDD-related *International Classification of Diseases, 9*^*th*^*revision – Clinical Modification* (ICD-9-CM) diagnosis codes (296.2x, 296.3x), ≥ 1 anti-depressant prescription, ≥ 1 mention of MDD diagnoses within inpatient or outpatient clinical notes, and ≥1 9-item Patient Health Questionnaire (PHQ-9) score of 15 or higher. The National Drug File – Reference Terminology (NDF-RT) [19] was used to identify prescriptions for antidepressants (C8870), including tricyclic antidepressants (C8872), monamine oxidase inhibitor antidepressants (C8874), or other antidepressants (C8876). Patients with diagnoses of bipolar disorders (ICD-9-CM: 296.1x, 296.4x – 296.9x), dementia/delirium (ICD-9-CM: 290.x), or psychotic disorders (ICD-9-CM: 295.x, 298.x) were excluded. The index date was defined as the first day each subject met the EHR-based algorithm definition of MDD. To eliminate prevalent MDD cases, MDD cases were restricted to those with no evidence of a MDD diagnosis prior to their index date in their EHR records.

### Control subjects

Each MDD case identified by the EHR algorithm was paired with one control subject with no evidence of MDD-related records (listed above) in their EHR. Controls also could not have had prior diagnoses of schizophrenia, related primary psychotic disorders, or manic depressive illness/bipolar disorders. The control subjects were matched 1:1 with MDD cases on birth year (± 2 years), sex, and having at least one outpatient clinic visit during the same study year. The index date for control subjects was defined as the date of the first clinic visit within the index study year.

### Chronic conditions

All ICD-9-CM diagnosis codes (outpatient or inpatient) were searched electronically for all subjects, using the 5 years preceding the index dates. Although the EHR data were available since 1994, the year 2000 was chosen as the starting point for MDD ascertainment in order to provide at least a 5 year ascertainment period for identifying chronic conditions. These ICD-9-CM codes were used to define 24 chronic condition categories defined by the Chronic Conditions Data Warehouse (https://www.ccwdata.org/web/guest/condition-categories), after removing dementia/Alzheimer’s disease and depression. To reduce false-positive misclassification of the chronic conditions, only persons who received at least two diagnostic codes for a given condition separated by > 30 days were considered to have that condition [8, 16].

### Additional data

Both obesity and socioeconomic deprivation have been associated with multimorbidity involving both physical and mental health conditions [20–22]. Therefore, we included them in the analysis when quantifying the impact of chronic conditions on MDD. Obesity was defined using the following ICD-9-CM codes: 278.0, 564.2, V45.3, V45.86, 649.1, 649.2, V85.3, V85.4, and V77.8. We were unable to ascertain a direct measure of socioeconomic status. Instead, we used level of educational attainment as a proxy for socioeconomic status. The highest level of education was self-reported from a questionnaire administered to patients on at least a yearly basis and categorized as high school or less, some college, 4-year college degree, and post-graduate studies.

### Statistical analysis

Demographic characteristics of incident MDD cases and matched controls were summarized using proportions. The number of chronic conditions and frequency of each chronic condition were also presented, separately for MDD cases and controls. Proportions for prostate cancer and benign prostatic hyperplasia were calculated for men only, while proportions for endometrial cancer were calculated for women only.

For each stratum defined by age at index date (≤ 45 years, 46 – 60 years, and over 60 years), conditional logistic regression models were applied to assess the association of each chronic condition with risk of subsequent MDD diagnosis, adjusting for educational attainment and obesity. Odds ratios (ORs) and corresponding 95 % confidence intervals (CIs) were presented. Gradient boosting machine (GBM) models (including sex interaction) were applied to quantify the relative influence (RI) of each chronic condition on the risk of incident MDD diagnosis in presence of other conditions as well as educational attainment and obesity. The GBM modeling approach is a machine learning technique for building a multivariable prediction model by incorporating all of the variables without variable selection [23, 24]. RI is a measure of a given variable’s importance, relative to that of other variables, in the model prediction process. The measure is based on the number of times a variable is selected for splitting in a decision tree, weighted by the improvement of the model fitting as a result of the split and further standardized so that the sum of RI from all variables adds up to 100 % [23–25]. For this analysis, the higher the RI value of a given chronic condition, the more important it is for the risk of incident diagnosed MDD, relative to the other conditions included in the model. Within each of the three age groups, chronic conditions occurring in at least 1 % of the patients were analyzed. To assess the impact of multiple chronic conditions, the GBM models were applied with and without including the total disease burden (the number of total chronic conditions among 24 conditions). For all analyses, we used R free statistical software (http://cran.r-project.org) and related packages including the *gbm* package.

## Results

### Demographic and clinical characteristics of the subjects

Among all patients receiving primary care at Mayo Clinic during the study period, a total of 11,375 patients met the definition of MDD during the study period. The majority of patients were Caucasian; the median age at MDD diagnosis was 43 years and 65 % were female (Table 1). Slightly greater than 30 % of these patients had an education level of high school degree or less. MDD cases and non-MDD controls were well-matched on age and sex.

**Table 1:** Characteristics of MDD patients and age-sex-matched controls

	MDD cases (*n* = 11,375)	Matched controls (*n* = 11,375)
Age (years) at index date, *N* (%)		
45 or younger	6215 (54.6 %)	6218 (54.7 %)
Between 46 and 60	2732 (24.0 %)	2737 (24.1 %)
Over 60	2528 (21.3 %)	2420 (21.3 %)
Sex, female *N* (%)	7424 (65.3 %)	7423 (65.3 %)
Race, white *N* (%)	10,011 (88.0 %)	10,706 (94.1 %)
Education at index date		
High school or less	3496 (30.7 %)	3708 (32.6 %)
Some college	3341 (29.4 %)	3788 (33.3 %)
College degree (4-year)	1779 (15.6 %)	1653 (14.5 %)
Post-graduate	1821 (16.0 %)	1383 (12.2 %)
Unknown	938 (8.3 %)	843 (7.4 %)
Diagnosed obesity, *N* (%)	699 (6.2 %)	382 (3.4 %)
Number of chronic conditions, one or more *N* (%)		
Overall	4741 (41.2 %)	3632 (31.9 %)
45 or younger^a^	1541 (24.8 %)	957 (15.4 %)
Between 46 and 60^a^	1360 (49.8 %)	1031 (37.7 %)
Over 60^a^	1840 (75.8 %)	1644 (67.9 %)

^a^Frequencies were calculated using the number of subjects in each age group as the denominator

Over 40 % of the incident MDD cases and slightly under one-third of controls had at least one chronic condition during the 5 years preceding the index dates (Table1). For both MDD cases and non-MDD controls, the frequency of having at least one chronic condition increased with age. Within each age stratum, the frequency was higher for MDD cases compared controls (Table 1).

**Fig. 1:**
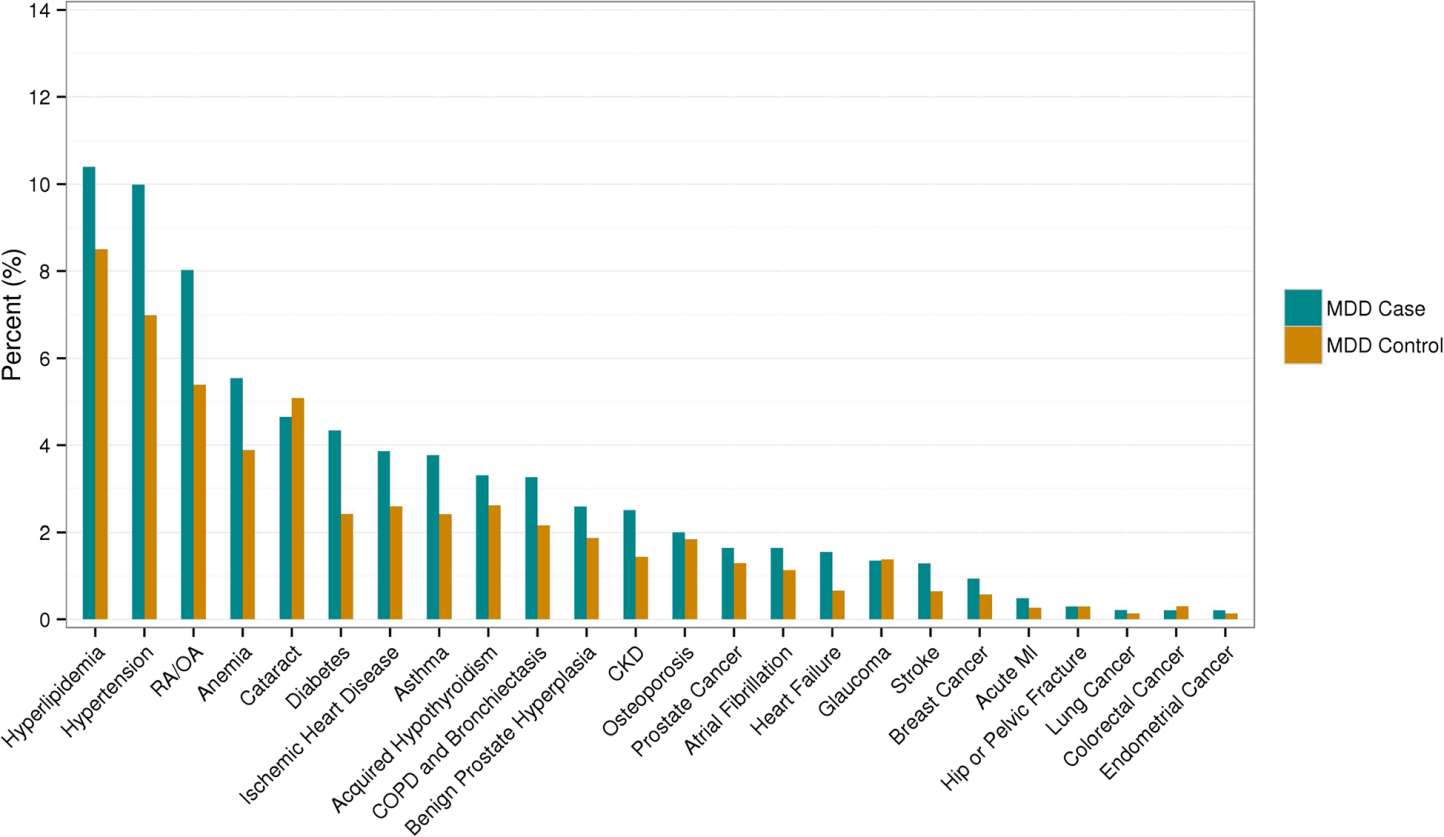
Frequency of chronic conditions occurred prior to index dates, separately by cases and controls. Proportions for prostate cancer and benign prostate hyperplasia were among men and endometrial cancer was among women

The most prevalent chronic conditions among MDD cases and non-MDD controls, regardless of age and sex, are shown in Fig. 1. Of the 24 chronic conditions considered, hyperlipidemia was the most common in both cases and controls (10.4 % vs 8.5 %), followed by hypertension (10.0 % vs 7.0 %) and rheumatoid arthritis/osteoarthritis (RA/OA, 8.0 % vs 5.4 %). The frequencies of nearly all chronic conditions (except for eye conditions such as glaucoma and cataracts) were higher for the cases compared to controls.

### Association of chronic conditions with the risk of incident MDD

**Fig. 2:**
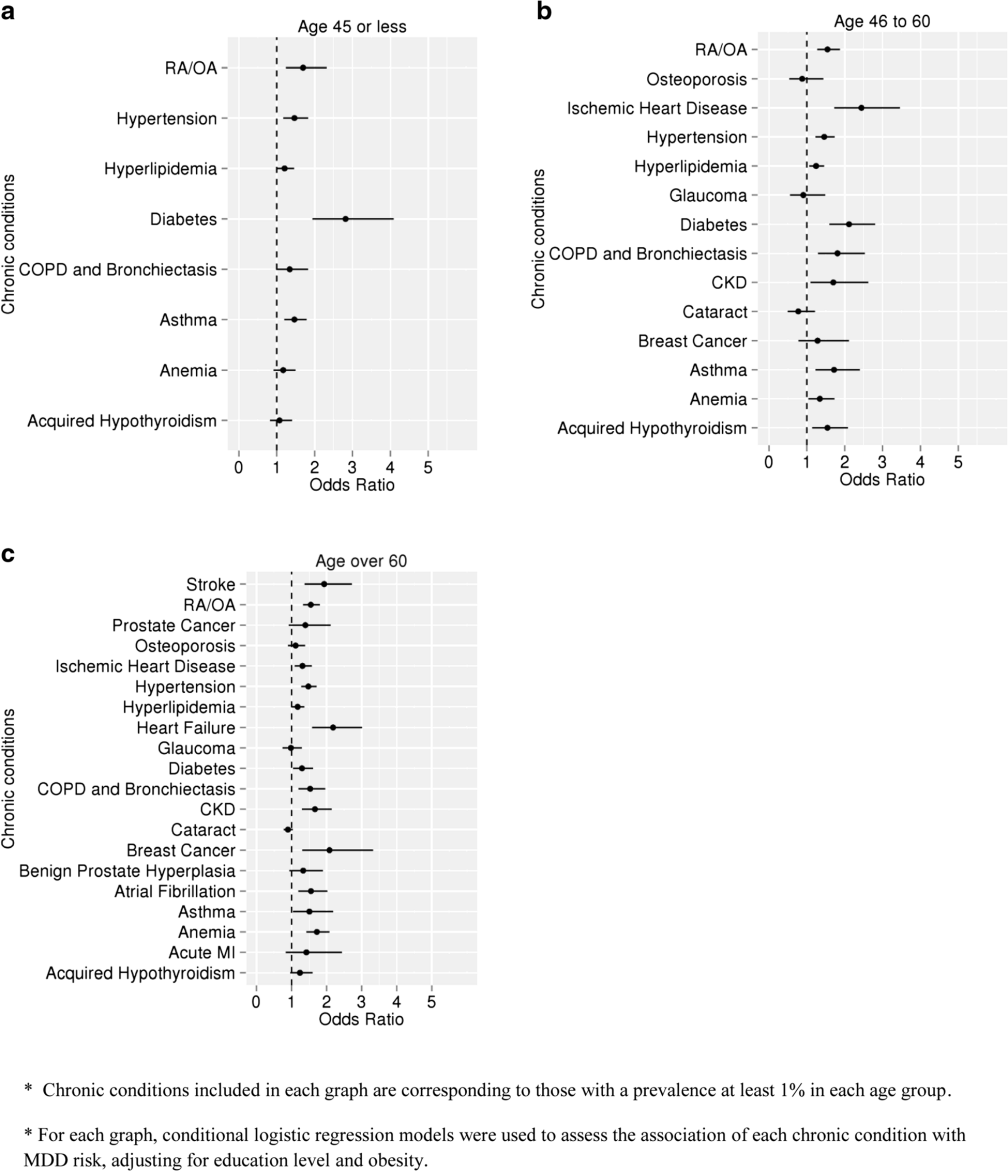
Association of each chronic condition with the risk of MDD, stratified by different age group and adjusted by education attainment and obesity. Three different age groups were used: subjects aged 45 or younger (**a**), subjects aged between 46 and 60 (**b**), and subjects aged 61 or older (**c**)

Adjusting for educational attainment and obesity, the association of each chronic condition with the risk of incident MDD diagnosis, stratified by age groups, is shown in Fig. 2(a) through (c). In the youngest age stratum (≤ 45 years), eight out of 24 chronic conditions occurred in at least 1 % of the study subjects. Compared to controls, MDD cases had significantly higher rate of diabetes mellitus (DM, 1.8 % vs. 0.6 % OR 2.8, 95 % CI 1.9 – 4.1), RA/OA (1.8 % vs. 1.0 %, OR = 1.7, 95 % CI 1.2 – 2.3), hypertension (3.5 % vs. 2.2 %, OR 1.5, 95 % CI 1.2 – 1.8), and asthma (4.1 % vs. 2.7 %, OR = 1.5, 95 % CI 1.2 – 1.9). No statistically significant between-group differences in the rates of hypothyroidism, chronic obstructive pulmonary disease, or anemia were observed.

In the 46–60 years old age stratum, 14 of the 24 chronic conditions occurred in at least 1 % of study subjects. Subjects with MDD had significantly higher rates of ischemic heart disease (4.3 % vs 2.0 %, OR 2.4, 95 % CI 1.7 – 3.5), DM (6.3 % vs 2.2 %, OR 2.1, 95 % CI 1.6 – 2.8), asthma (3.7 % vs 2.0 %, OR = 1.7, 95 % CI 1.2 – 2.4), and RA/OA (10.9 % vs 7.6 %, OR = 1.5, 95 % CI 1.2 – 1.9) compared to controls. There were no statistically significant differences in rates of osteoporosis, glaucoma, cataracts, or breast cancer.

For the oldest age stratum (>60 years), 20 of the 24 chronic conditions occurred in at least 1 % of study subjects. Stroke (4.1 % vs 2.2 %, OR 1.9, 95 % CI 1.4 – 2.7) and heart failure (5.3 % vs 2.4 %, OR 2.2, 95 % CI 1.6 – 3.0) were strongly associated with MDD. The strength of association between RA/OA and MDD among persons aged over 60 years was similar to those of other age groups (20.6 % vs 14.1 %, OR 1.5, 95 % CI 1.3 – 1.8).

### Relative contribution of chronic conditions to MDD risk in the presence of education and obesity

**Fig. 3:**
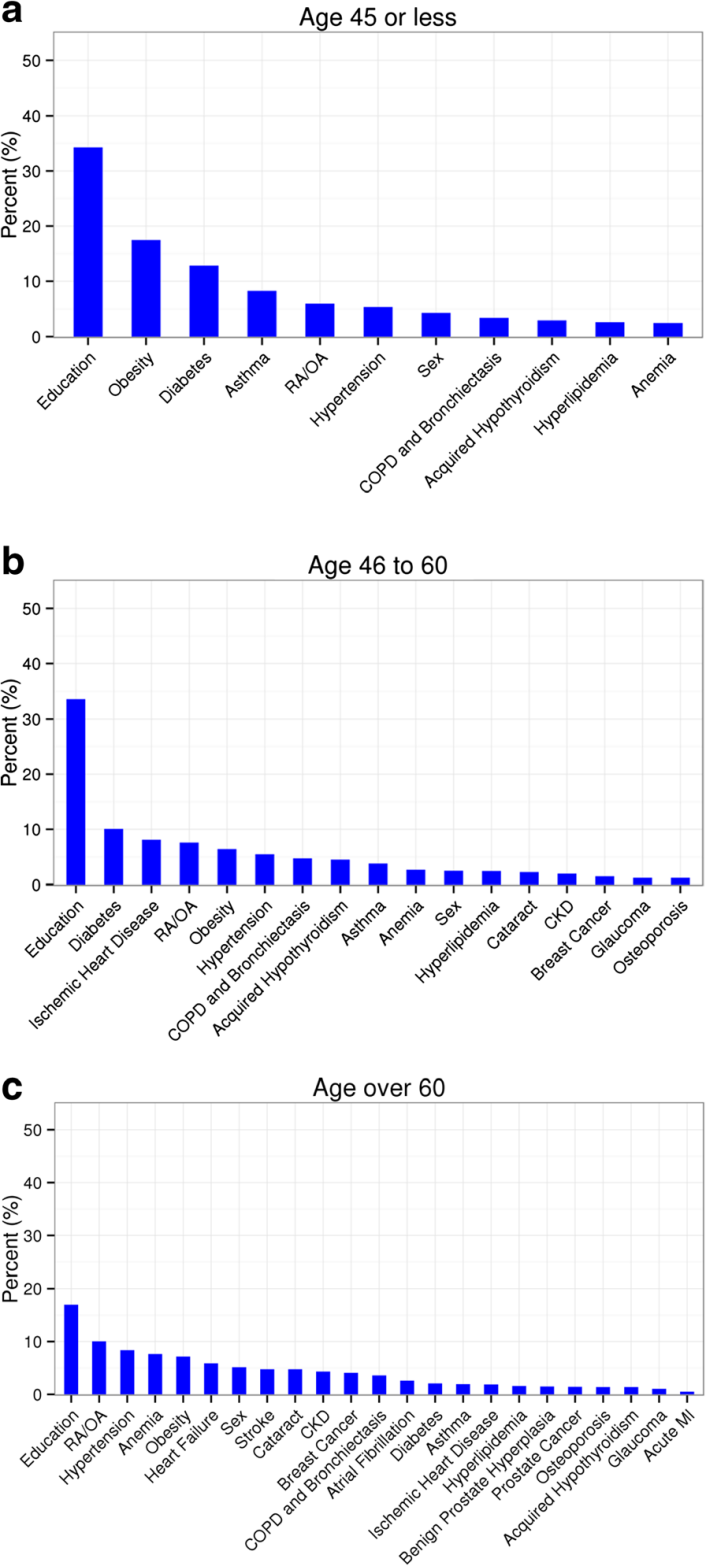
Relative influence (%) of chronic conditions on MDD risk, stratified by different age group. Three different age groups were used: subjects aged 45 or younger (**a**), subjects aged between 46 and 60 (**b**), and subjects aged 61 or older (**c**)

Importance of each chronic condition on the risk of incident MDD diagnosis varied according to age stratum (Fig. 3). The most influential factor for all age strata was educational attainment (RI = 34 %, 34 %, and 17 % for subjects aged ≤ 45, between 46 and 60, and over 60 years, respectively). Among the 24 chronic conditions that were the main focus of this study, DM showed the greatest contribution to MDD risk for those aged 60 years or younger (RI of 13 % and 10 % for subjects aged ≤ 45 years, and 46 – 60 years, respectively). In the oldest age group (aged over 60 years), the greatest contribution to MDD risk was observed for RA/OA (RI of 10 %). Compared to its significant contribution in younger age group (≤ 60 years), DM showed little influence in older subjects (RI = 2.1 %).

When the total disease burden (total number of comorbid conditions) was also considered for quantifying relative contribution of individual somatic conditions to MDD risk, all chronic conditions showed minimal influence regardless of age (RI less than 10 %), while the total disease burden showed the greatest contribution (RI = 50 %, 42 %, and 36 % for subjects aged ≤ 45 years, between 46 and 60, and over 60 years, respectively). Educational attainment still showed a relatively large contribution in the presence of the total disease burden for MDD risk (RI = 21 %, 28 %, and 17 % for subjects aged ≤ 45 years, between 46 and 60, and over 60 years, respectively).

## Discussion

Epidemiological studies have documented strong and significant associations between prevalent MDD and several somatic health conditions [20, 26–30]. Most of this literature has consisted of cross-sectional studies, or of longitudinal studies focused primarily on single chronic condition pairings with MDD. The results of cross-sectional studies are limited in that documented associations between MDD and chronic conditions could reflect any of a number of combinations including (1) the incidence of chronic conditions in patients with MDD, (2) the incidence of MDD in people with specific health conditions, (3) the effect of MDD and specific conditions on the prognosis and mortality associated with one another [26]. Studies focused on MDD associations with single chronic health conditions are also limited in that the relative influence of the conditions of interest on the risk of MDD are often not considered in the context of other comorbid physical health conditions. Consistent with the current literature, we observed that almost all chronic conditions considered showed some level of association with MDD risk when considered individually, even after accounting for educational attainment. When considering all chronic conditions simultaneously, we found that the greatest relative influence for MDD risk was observed for diabetes mellitus among subjects aged 60 years or younger and RA/OA for subjects over 60 years. We also observed that the total disease burden (measured as the number of chronic conditions) was the greatest contributor for MDD risk, followed by socioeconomic status (using education attainment as a surrogate). However, this study focused on identifying individual chronic conditions with the greatest influence on MDD risk, as it is difficult to implement clinical recommendations based on general disease burden without also targeting co-occurring chronic somatic conditions individually.

Although no single chronic condition had a very large effect on the risk of MDD diagnosis, our findings support existing recommendations for MDD screening when adequate resources for follow-up diagnosis and treatment are available, and highlight the importance of collaborative depression care, especially for patients who are diagnosed with the physical health conditions that may have greater relative importance in terms of MDD risk. This is particularly important in light of evidence that most patients with chronic medical conditions do not receive proper diagnosis and treatment of psychiatric comorbidities [30].

In our study, diabetes had the greatest relative contribution to depression in non-elderly adults even after accounting for obesity. Epidemiological studies have long documented an association between depression and higher rates of type 1 and type 2 diabetes mellitus in adults. In an influential meta-analysis of 20 controlled epidemiological studies, the risk of depression was twice as high among persons with prevalent diabetes relative to nondiabetic controls [31]. The association between depression and diabetes may be particularly strong for persons with type 2 diabetes mellitus, although several prior studies failed to control for several variables associated with increased risk of clinically significant depression [32]. Numerous models have been proposed in an attempt to provide mechanistic explanations for the high rates of depression-diabetes co-occurrence. These include negative lifestyle factors and higher rates of overweight and obesity among depressed individuals that increase the risk of new-onset type 2 diabetes, poorer adherence to diabetes treatment among depressed relative to non-depressed persons, and orexigenic effects of some antidepressant medications [33–36]. Additionally, a number of shared biological mechanisms have been hypothesized, including chronic hypothalamic-pituitary adrenal (HPA) axis dysfunction leading to abnormal glucocorticoid signaling, chronic inflammation, disruption of normal circadian rhythms, and abnormalities in neurotrophin and monamine signaling [37, 38]. The identification of targets for primary prevention of both type 2 diabetes and depressive disorders based on the ascertainment of shared etiological mechanisms is a current research priority [39].

Among subjects aged over 60 years, a significant contribution to the risk of depression was made by RA/OA. As is the case with diabetes, epidemiological studies have documented high rates of depressive disorder comorbidity among patients with RA [40], even after controlling for the overlap in symptomatology between the two conditions [41]. Pain and disability from RA has been proposed as one clinical factor that may increase the risk of comorbid depression, which may in turn worsen clinical outcome related to RA [42]. More recent studies have focused on shared biological factors between depression and RA—specifically, the systemic inflammation that is characteristic of RA and other inflammatory disorders may also have a role in the development of depression, even among persons without RA [43].

Our study has several limitations. First, case ascertainment for MDD and the chronic conditions under consideration was conducted solely based on information from EHRs. Although our validated EHR-based algorithm used multiple data sources (billing codes, clinical notes, and medication use) to identify MDD cases, neither the MDD cases nor the chronic conditions in this study were subject to confirmatory medical record review. We also acknowledge that the information recorded in the medical records may not be accurate or complete enough for a confirmatory diagnosis of MDD. Second, medical records prior to 1994 were not utilized in this study, as they were not available through our EHR system. Therefore, it is possible that a proportion of depression episodes occurred prior to 1994 would have been missed, which potentially impacts our study findings. Third, high rates of missed depression diagnoses in clinical practice have been well supported in literature. Thus, a proportion of controls may have undiagnosed depression, which may have reduced statistical power to distinguish comorbidity patterns between cases and controls. Fourth, diagnostic practice may have changed during the study period (between 2000 and 2013). This could potentially have affected the rates of diagnosis of MDD and co-occurring somatic conditions. Fifth, the severity of MDD was not incorporated in the current study, an important consideration given evidence that MDD severity may correlate with the severity of specific somatic conditions or the number of co-occurring somatic conditions [44–48]. Sixth, the majority of our study population was Caucasian; thus, our results should be replicated in more diverse populations. As a related point, the mean age of our cohort is younger than some other studies of multimorbidity in primary care populations [49]. For most patients, MDD has a relatively early age of onset, typically less than 40 years of age [13, 15]. Therefore, persons in this age range seeking evaluation and treatment for MDD may be expected to present to their primary care physicians. This seems particularly likely if they have existing chronic medical conditions that are already being managed by their primary care physicians—a condition that would seem to apply to our study population. And finally, although education level has been used in prior research as a proxy for socioeconomic status [50], we were unable to adjust our results for more direct measures of socioeconomic status.

## Conclusion

 Our results suggest that specific chronic conditions such as diabetes mellitus and rheumatoid arthritis/osteoarthritis may have greater influence than others on the risk of MDD. Ethics approval and consent to participate.

This study utilizing secondary EHR data was reviewed and approved by the Mayo Clinic Institutional Review Board (IRB).

### Consent for publication

Not applicable

### Availability of data and materials

The data cannot be shared due to confidentiality issues.

## Abbreviations

CI: confidence interval
DM: diabetes mellitus
EHR: electronic health record
GBM: gradient boosting machine
ICD-9-CM: international classification of diseases, 9^th^ revision – clinical modification
MDD: major depressive disorder
OR: odds ratio
RA/OA: rheumatoid arthritis/osteoarthritis
RI: relative influence

## References

[1] Kessler RC, Birnbaum H, Bromet E, Hwang I, Sampson N, Shahly V (2010). Age differences in major depression: results from the National Comorbidity Survey Replication (NCS-R). Psychol Med.

[2] Kessler RC, McGonagle KA, Swartz M, Blazer DG, Nelson CB (1993). Sex and depression in the National Comorbidity Survey I: Lifetime prevalence, chronicity and recurrence. J Affect Disord.

[3] Murray CJL, Lopez AD (2013). Measuring the Global Burden of Disease. N Engl J Med.

[4] Kupfer DJ, Frank E, Phillips ML (2012). Major depressive disorder: new clinical, neurobiological, and treatment perspectives. Lancet.

[5] Hare DL, Toukhsati SR, Johnsson P, Jaarsma T (2014). Depression and cardiovascular disease: a clinical review. Eur Heart J.

[6] Walker J, Hansen CH, Martin P, Symeonides S, Ramessur R, Murray G, Sharpe M (2014). Prevalence, associations, and adequacy of treatment of major depression in patients with cancer: a cross-sectional analysis of routinely collected clinical data. Lancet Psychiatr.

[7] Anderson RJ, Freedland KE, Clouse RE, Lustman PJ (2001). The prevalence of comorbid depression in adults with diabetes. Diabetes Care.

[8] Rocca WA, Boyd CM, Grossardt BR, Bobo WV, Finney Rutten LJ, Roger VL, Ebbert JO, Therneau TM, Yawn BP, St Sauver JL (2014). Prevalence of multimorbidity in a geographically defined American population: patterns by age, sex, and race/ethnicity. Mayo Clin Proc.

[9] Koike AK, Unutzer J, Wells KB (2002). Improving the care for depression in patients with comorbid medical illness. Am J Psychiatry.

[10] Hays JC, Krishnan KR, George LK, Pieper CF, Flint EP, Blazer DG (1997). Psychosocial and physical correlates of chronic depression. Psychiatry Res.

[11] Muskin PR (2010). Major depressive disorder and other medical illness: a two-way street. Ann Clin Psychiatry.

[12] Aina Y, Susman JL (2006). Understanding comorbidity with depression and anxiety disorders. J Am Osteopath Assoc.

[13] Eaton WW, Anthony JC, Gallo J (1997). Natural history of DIS/DMS major depression: the Baltimore ECA followup. Arch Gen Psychiatry.

[14] Weiss B, Garber J (2003). Developmental differences in the phenomenology of depression. Dev Psychopathol.

[15] Kessler RC, Berglund P, Demler O, Jin R, Merikangas KR, Walter EE (2005). Lifetime prevalence and age-of-onset distributions of DMS-IV disorders in the National Comorbidity Survey Replication. Arch Gen Psychiatry.

[16] St Sauver JL, Boyd C, Grossardt BR, Bobo WV, Finney Rutten LJ, Roger VL, Ebbert JO, Therneau TM, Yawn BP, Rocca WA (2015). Risk of developing multimorbidity across all ages in an historical cohort study: differences by sex and ethnicity. BMJ Open.

[17] Pathak J, Simon G, Li D, Biernacka JM, Jenkins GJ, Chute C, Hall-Flavin DK, Weinshilboum RM (2014). Detecting association between major depressive disorder treatment and essential hypertension using electronic health records. AMIA Jt Summits Transl Sci Proc.

[18] Bielinski SJ, Chai HS, Pathak J, Talwalkar JA (2011). Mayo Genome Consortia: A genotype-phenotype resource for genome-wide association studies with an application to the analysis of circulating bilirubin levels. Mayo Clin Proc.

[19] Pathak J, Chute CG (2010). Analyzing categorical information into two publically available drug terminologies: RxNorm and NDF-RT. J Am Inform Assoc.

[20] Katon WJ (2011). Epidemiology and treatment of depression in patients with chronic medical illness. Dialogues Clin Neurosci.

[21] Neeleman J, Ormel J, Bijl RV (2001). The distribution of psychiatric and somatic III health: associations with personality and socioeconomic status. Psychosom Med.

[22] Barnett K, Mercer SW, Norbury M, Watt G, Wyke S, Guthrie B (2012). Epidemiology of multimorbidity and implications for health care, research, and medical education: a cross-sectional study. Lancet.

[23] Ridgeway G. Generalized boosted models: a guide to the gbm package. Available at http://cran.rproject.org/web/packages/gbm/vignettes/gbm.pdf.

[24] Atkinson EJ, Therneau TM, Melton LJ 3^rd^, Camp JJ, Achenbach SJ, Amin S, Khosla S. Assessing fracture risk using gradient boosting machine (GBM) models. J Bone Miner Res. 2012; doi; 10.1002/jbmr.1577. 10.1002/jbmr.1577 PMC3408850 22367889

[25] Natekin A, Knoll A (2013). Gradient boosting machines, a tutorial. Front Neurobot.

[26] Patten SB, Beck CA, Kassam A, Williams JV, Barbui C, Metz LM (2005). Long-term medical conditions and major depression: strength of association for specific conditions in the general population. Can J Psychiatry.

[27] Ali S, Stone MA, Peters JL, Davies MJ, Khunti K (2006). The prevalence of comorbid depression in adults with Type 2 diabetes: a systematic review and meta-analysis. Diabet Med.

[28] Schleifer SJM-HM, Coyle DA, Slater WR, Kahn M, Gorlin R, Zucker HD (1989). The nature and course of depression following myocardial infarction. Arch Intern Med.

[29] Spijkerman T, de Jonge P, van den Brink RH, Jansen JH, May FJ, Crijns HJ, Ormel J (2005). Depression following myocardial infarction: first-ever versus ongoing and recurrent episodes. Gen Hosp Psychiatry.

[30] Mikkelsen RL, Middelboe T, Pisinger C, Stage KB (2004). Anxiety and depression in patients with chronic obstructive pulmonary disease (COPD). A review. Nord J Psychiatry.

[31] Anderson RJ, Freedland KE, Clouse RE, Lustman PJ (2001). The prevalence of comorbid depression in adults with diabetes: a meta-analysis. Diabetes Care.

[32] Berge Ll, Riise T. Comorbidity between type 2 diabetes and depression in the adult population: Directions of the association and its possible pathophysiological mechanisms. Int J Endocrinol. 2015;2015:164760. doi:10.1155/2015/164760. 10.1155/2015/164760 PMC4589629 26457080

[33] Barnard K, Peveler RC, Holt RI (2013). Antidepressant medication as a risk factor for type 2 diabetes and impaired glucose regulation: systematic review. Diabetes Care.

[34] Berk M, Williams LJ, Jacka FN (2013). So depression is an inflammatory disease, but where does the inflammation come from?. BMC Med.

[35] Bruce DG, Davis WA, Starkstein SE, Davis TM (2005). A prospective study of depression and mortality in patients with type 2 diabetes: the Fremantle Diabetes Study. Diabetologia.

[36] Katon W, von Korff M, Ciechanowski P (2004). Behavioral and clinical factors associated with depression among individuals with diabetes. Diabetes Care.

[37] Krabbe KS, Nielsen AR, Krogh-Madsen R (2007). Brain-derived neurotrophic factor (BDNF) and type 2 diabetes. Diabetologia.

[38] Moulton CD, Pickup JC, Ismail K (2015). The link between depression and diabetes: the search for shared mechanisms. Lancet Diabetes Endocrinol.

[39] Eckel RJ, Kahn SE, Derrannini E (2011). Obesity and type 2 diabetes: What can be unified and what needs to be individualized?. J Clin Endocrinol Metab.

[40] Cutolo M, Kitas GD, van Riel PL (2014). Burden of disease in treated rheumatoid arthritis patients: going beyond the joint. Semin Arthritis Rheum.

[41] Dickens C, McGowan L, Clark-Carter D, Creed F (2002). Depression in rheumatoid arthritis: a systematic review of the literature with meta-analysis. Psychosom Med.

[42] Fishbain DA, Cutler R, Rosomoff HL, Rosomoff RS (1997). Chronic pain-associated depression: antecedent or consequence of chronic pain? A review. Clin J Pain.

[43] Dantzer R, O’Connor JC, Freund GG, Johnson RW, Kelley KW (2008). From inflammation to sickness and depression: when the immune system subjugates the brain. Nat Rev Neurosci.

[44] Lesser IM, Leuchter AF, Trivedi MH, Davis LL, Wisniewski SR, Balasubramani GK, Petersen T, Stegman D, Rush AJ (2005). Characteristics of insured and noninsured outpatients with depression STAR(*)D. Psychiatr Serv.

[45] Frasure-Smith N, Lesperance F, Habra M, Talajic M, Khairy P, Dorian P, Roy D, Atrial Fibrillation and Congestive Heart Failure Investigators (2009). Elevated depression symptoms predict long-term cardiovascular mortality in patients with atrial fibrillation and heart failure. Circulation.

[46] Imran MY, Saira Khan EA, Ahmad NM, Farman Raja S, Saeed MA, Ijaz HI (2015). Depression in rheumatoid arthritis and its relation to disease activity. Pak J Med Sci.

[47] Stein PK, Carney RM, Freedland KE, Skala JA, Jaffe AS, Kleiger RE, Rottman JN (2000). Severe depression is associated with markedly reduced heart rate variability in patients with stable coronary heart disease. J Psychosom Res.

[48] Tang ST, Chen JS, Chou WC, Lin KC, Chang WC, Hsieh CH, Wu CE (2016). Prevalence of severe depressive symptoms increases as death approaches and is associated with disease burden, tangible social support, and high self-perceived burden to others. Support Care Cancer.

[49] Violan C, Foquet-Boreu Q, Flores-Mateo G, Salisbury C, Blom J, Freitag M, Glynn L, Muth C, Valderas JM (2014). Prevalence, determinants and patterns of multimorbidity in primary care: a systematic review of observational studies. PLoS ONE.

[50] Braveman PA, Cubbin C, Egerter S, CHideya S, Marchi KS, Metzler M, Posner S (2005). Socioeconomic status in health research: one size does not fit all. JAMA.

